# 

**Published:** 1892-03

**Authors:** 


### CONTENTS:

ORIGINAL ARTICLES: PAGE

Notes on Parasites. By Charles W. Stiles, Ph.D. .... 143
Calcified Fibrous Nodes of the Liver and Lungs of the Horse. By S. J. J. Harger, V. M. D. .... 149
Contribution to the Study of Mallein. By Dr. W. Dieckerhoff and Dr. R. Lothes. 160
Actinomycosis. By Dr. R. W. Hickman and Dr. Victor A. Norgaard .... 161
The Committee Reports on Meat Inspection and Actinomycosis. By Olaf Schwarzkopff .... 171
Actinomycosis and U. S. Meat Inspection. By W. L. Williams, V. S .... 177
Successful Aponeurotomy in Springhalt. By S. J. J. Harger, V.M.D .... 180
Tuberculosis in Giraffe. By J. C. Meyers, Sr., V.S. .... 181
Retention of a Dead Fœtus by a Cow. By D. P. Frame .... 182

SELECTIONS:

Variola and Vaccine .... 184
Foot and Mouth Disease .... 185
Recent Patents .... 186

EDITORIAL:

United States Army Veterinary Service .... 189
Scientific Honors .... 191
United States Veterinary Medical Association .... 192
Necrology. Sir William Macleay, Kt. F. L. S., M. L. C .... 192

Antiseptic Dressing of Wounds in the Lower Animals. By R. W. Burke, F.R.C.V.S. .... 193

SOCIETY PROCEEDINGS:

United States Veterinary Medical Association .... 201
Massachusetts Veterinary Association .... 202
Western Iowa Veterinary Medical Association .... 203
Ontario Veterinary College Medical Society .... 204
Maryland State Veterinary Medical Association—Special Meeting .... 505
Maryland State Veterinary Medical Association—Sixth Annual Meeting .... 205

REVIEWS .... 206

